# Human Defence Factors in Different Gestational Week Placenta: A Pilot Study

**DOI:** 10.3390/life15010086

**Published:** 2025-01-13

**Authors:** Andris Kamergrauzis, Mara Pilmane, Anna Junga

**Affiliations:** Institute of Anatomy and Anthropology, Riga Stradins University, Kronvalda Boulevard 9, LV-1010 Riga, Latvia; a.kamergrauzis@gmail.com (A.K.); anna.junga@rsu.lv (A.J.)

**Keywords:** placenta, gestational week, defence factors, immunohistochemistry

## Abstract

Background: Numerous studies have shown the presence of multiple defence factors in placental tissue, although their role is partially understood; therefore, the aim of this study was to evaluate the expression of nuclear factor-kappa B (NF-κB); human beta-defensin 2, 3, and 4 (HBD-2,3,4); cathelicidine (LL-37); heat shock protein 60 (HSP60); and interleukin 10 (IL-10) in dissimilar gestational week placental tissue and display correlations between immunoreactive cells. Methods: A total of 15 human placental tissue samples were acquired from mothers with different gestational weeks: 28, 31, and 40. Routine staining and immunohistochemistry for the samples were executed. The evaluation of data was performed with semi-quantitative methods, and, for statistical analysis, the Kruskal–Wallis test was used. Spearman’s rank correlation was used for calculating correlations. Results: NF-κB, HBD- 2,3,4, HSP60, and IL-10 expression were discovered in every examined placental tissue cell type. LL-37 expression was found only in Hofbauer cells. A rise in expression with higher gestational weeks was noted in LL-37-positive Hofbauer cells (*p* = 0.03), HBD-3-positive cytotrophoblasts (*p* = 0.007), endothelial cells (*p* = 0.024), extraembryonic mesodermal cells (*p* = 0.004), and HBD-4-positive endothelial cells (*p* = 0.001). Numerous statistically significant moderate and strong positive correlations between defence factors were discovered. Conclusions: The persistence of Hofbauer cell accumulations underlines the growing significance of placental macrophages in placental protection. The expression of positive defence factors and a rise in expression in tissue protection factors (HBD-3, LL-37, HBD-4) in higher gestational weeks may indicate these factors as the most significant protectors of the placenta in ontogenetic aspects. The high number of statistically significant positive and negative correlations between positive cells show a strong network to sustain distressed placental growth and therefore pregnancy.

## 1. Introduction

The placenta is an important transitional fetal organ and plays a significant role in the health of the fetus and its mother. The placenta sustains fetus growth by delivering oxygen, nutrients, and hormones; moreover, it is also responsible for removing waste products. Flawed early placental development is the main cause of frequent disorders during pregnancy, including fetal growth restriction, pre-eclampsia, stillbirth, and recurrent miscarriage. In addition, poor pregnancy conditions affect the life-long health of the fetus [1,2,3].

The placenta acts as a physical, selective, and protective barrier between fetal and maternal circulation, stopping the possible transfer of different pathogens. Significant factors in this barrier are trophoblasts, and strong evidence reveals that trophoblasts function in organizing signals to optimize transport functions, hormone production in the placenta, and immunological defence mechanisms for the developing fetus [3]. Blood vessels, macrophages such as Hofbauer cells, extraembryonic mesodermal cells, and cytotrophoblasts have a role in protective development mechanisms to sustain healthy placenta function and therefore pregnancy [4,5,6,7].

Multiple tissue factors belong to the local protection systems in the placenta, although they have not been researched enough under different placental developmental times. Cathelicidins are a family of host defence peptides, and they are conserved and play an important role in innate immunity [8]. Cathelicidin’s (LL-37) C-terminal of this protein contains a 37-amino-acid-long peptide starting with two Leu residues, which shows broad antibacterial activity [8,9,10]. LL-37 protein is produced in neutrophils, T cells, natural killer (NK) cells, mast cells, and many other types of tissue cells. LL-37 can also be found in amniotic fluid [11]. LL-37 has multiple functions, including antimicrobial activity against multiple types of microorganisms. It plays a role in organizing the immune response towards infection, locally modulates inflammation, and promotes angiogenesis [8,12].

Nuclear factor kappa B (NF-κB) is a protein transcription factor, and it is considered to be a regulator of innate immunity [13,14]. The functions of NF-κB include regulating multiple pathways that can impact cellular function, including proliferation, differentiation, apoptosis, angiogenesis, epithelial-to-mesenchymal transition, and oxidative stress [15,16,17,18].

Human β Defensin 2 (hBD-2) is a cysteine-rich, cationic, low molecular weight antimicrobial peptide, showing its active antimicrobial activity [19]. hBD-2 is induced by inflammation and the infection of various microorganisms [20]. Human β-Defensins 2 are produced by epithelial cells of the chorionic membrane, amniotic membrane, and vaginal wall, and hBD-2 mRNA expression was noticed in placental, chorion, and villus cells [21,22,23]. High levels of hBD-2 in amniotic fluid have been reported in cases of preterm delivery, but a decrease in hBD-2 was noticed in patients with bacterial vaginosis during pregnancy; furthermore, hBD-2 is more active against gram bacteria [21,24], and hBD-2 could be a marker of intra-amniotic infections [22].

Human β defensin 3 (hBD-3) is a highly basic 45-amino-acid protein that acts both as an antimicrobial agent and as a chemoattractant molecule. It has broad-spectrum antibiotic activity against gram-negative and gram-positive bacteria and can show immunosuppressive activity as well [25,26,27,28]. hBD-3 mRNA expression is upregulated by treatment with inflammatory molecules that include IL-1β+TNFα, IFNγ, and phorbol ester [23]. The expression of hBD-3 in chorioamniotic membranes [29] and amnion and chorio-decidua of placental tissue has been found [30,31]. Furthermore, hBD-3 has been immunolocalized to the syncytiotrophoblast layer of term placental villi [30].

Human β defensin 4 (hBD-4) is a small positively charged cysteine-rich cationic polypeptide [32,33] that shows broad spectrum antimicrobial activity but compared with hBD1-3, it has low ionic strength [33]. There is expression of hBD-4 mRNA in the fetal membranes but, unlike hBD-1 and hBD-3, the expression of hBD-4 mRNA may not be totally affected by proinflammatory cytokine stimuli in chorioamniotic membranes [34].

Heat shock protein 60 (HSP60) are proteins that are chaperones and are mostly localized in the mitochondria of eukaryotic cells; they capture denatured substrate proteins in their central cavity [35,36,37]. HSP60 plays an important role in cell development, reproduction, thermoprotection, and immune defence [35,38]. HSP60 and other HSPs such as HSP90 and HSP70 are found to be localized in cytotrophoblasts, syncytiotrophoblasts, intermediate trophoblasts, Hofbauer, and endothelial cells [39]. Based on an immunostaining study, HSP60 was also immunolocalized in the decidual stromal cells during each trimester of pregnancy [39,40].

Interleukin 10 (IL-10) is a potent anti-inflammatory cytokine that plays a crucial and important role in preventing inflammatory and autoimmune pathologies [41]. IL-10 diminishes the production of inflammatory mediators and inhibits antigen presentation, although it enhances their uptake of antigens [41,42]. IL-10 regulates the differentiation and proliferation of several immune cells, including T cells, B cells, natural killer (NK) cells, antigen-presenting cells, mast cells, and granulocytes [43,44]. IL-10 expression has been found in placental villous trophoblasts, uterine NK cells (uNK cells), monocytes, and regulatory T cells in the decidua, and IL-10 receptors are localized to placental trophoblasts, decidual stromal cells, macrophages, and uterine NK cells (uNK cells) [43,45]. Alternatively activated macrophages (M2 macrophages) secrete high levels of the IL-10 cytokine upon polarization, and Hofbauer cells are thought to have an immunoregulatory phenotype consistent with being an alternatively activated macrophage (M2 macrophage); these cells can be stimulated by glucocorticoids and IL-10 while also secreting Il-10 [46,47].

Since the role of protective factors and their possible interaction in placental tissue is still not fully understood, the goal of this study was to discover the appearance and possible interaction of different defence factors in different developmental time placental tissues.

## 2. Materials and Methods

### 2.1. Subjects

The study was performed in accordance with the Helsinki Declaration in Latvia at Riga Stradiņs University Institute of Anatomy and Anthropology. Ethical committee at Riga Stradins University approved this research and permit was issued in March 2009 (12.03.2009 No. E-9 (2)). A total of 15 human placental tissue samples were obtained from 15 mothers with gestational weeks from 28 weeks to 40 weeks (Table 1). Mothers were aged 20 to 39 years with a number of graviditas ranging from I to VI. However, six females had one or multiple miscarriages earlier in their lives, resulting in the number of partus not matching the number of graviditas. Five placental tissue samples were obtained from mothers with delivery week 28, five placental tissue samples were from delivery week 31, and five placental tissue samples were from delivery week 40. A total of 10 placental tissue samples that were from week 28 and from week 31 can be classified as preterm delivery [48]. Eight of these deliveries were spontaneous preterm deliveries and two were noted as partum premature operationis. Five tissue samples from delivery week 40 were noted as partus mature with distress acuta. Inclusion criteria for tissue samples were delivery week.

### 2.2. Control Samples

A total of 5 human placental tissue control samples were obtained from 5 mothers with gestational age of 40 weeks. Mothers were aged from 27 to 42 years with a number of graviditas ranging from I to IV. The inclusion criteria for tissue samples was delivery week. Exclusion criteria were presence of any associated problem with pregnancy (Table 2).

### 2.3. Routine Morphological Assessment

Right after delivery, using single-use surgical knife, a cut was made and two 1 cm × 1 cm samples were acquired from symmetrically situated locations of the placentas, including every single layer of placental tissue. Acquired placental tissue samples were fixated for 24 h using 2% formaldehyde, 0.2% picric acid, and 0.1M phosphate buffer with a pH of 7.2. For 12 h, the samples were processed in Tyrode buffer, which contained 10% saccharose. Following this, the tissues were embedded into paraffin and cut into 5 µm sections. In addition to the morphological evaluation of placental tissue samples, hematoxylin and eosin staining was executed.

For immunohistochemical labelling the Biotin–Streptavidin method [49] was used to detect the following: NF-κB (ab7971 working dilution 1:100, Abcam, Cambridge, UK), HBD-2 (ab203077, working dilution 1:200, Abcam, Cambridge, UK), HBD-3 (orb183268, working dilution 1:100, Biorbyt, St Louis, MO, USA), HBD-4 (ab70215, working dilution 1:100, Abcam, Cambridge, UK), HSP-60 (sc-1052, working dilution 1:100, Santa Cruz Biotechnology, Dallas, TX, USA), Il-10 (ab134742, working dilution 1:50, Abcam, Cambridge, UK), LL-37 (orb88370, working dilution 1:100, Biorbyt LLC, St Louis, MO, USA).

### 2.4. Immunohistochemical (IHC) Analysis

For the evaluation of immunoreactive cells and their distribution, light microscopy and semi-quantitative non-parametric analysis were used in this study. Positively stained cells in placental tissue in the light microscope visual field were rated using a scale, which had the following labels: 0 no positive cells; 0/+ occasional (less than 10) positive cells; + few (10–15) positive cells; +/++ few to moderate (16–20) positive cells; ++ moderate (21–35) positive cells; ++/+++ moderate to numerous (36–50) positive cells; +++ numerous (51–70) positive cells; +++/++++ numerous to abundant (71–90) positive cells; ++++ abundant (more than 90) positive cells [50]. Positive placental tissue structures were observed in 5 visual fields, and rounded average was stated as the result of the specific sample. For all visual fields, magnification of x100 was used.

### 2.5. Statistical Analysis

To process obtained data from IHC analysis of positive cells in tissue samples, data were transformed into numerical form: 0: equals 0, 0/+: equals 0.5, +: equals 1, +/++: equals 1.5, ++: equals 2, ++/+++: equals 2.5, +++: equals 3, +++/++++: equals 3.5, ++++: equals 4. Kruskal–Wallis test was used to compare differences in factors based on placenta’s tissue age in distressed placental tissue sample group. The Mann–Whitney U test was used to assess if there were statistically significant differences in defence factor expression in placental structures between 40 gestational week control and distressed group. In addition, Spearman’s rank correlation coefficient was found and used to assess correlations between the chosen defence factors in distressed placental tissue sample group. Correlation with a value R < 0.2 counted as a very weak correlation, R = 0.20–0.39 expressed a weak correlation, R = 0.40–0.59 expressed a moderate correlation, R = 0.60–0.79 expressed a strong correlation, and 0.80–1.00 expressed a very strong correlation. Analyzing statistically significant differences in studied factors based on placental tissue age and correlations between the defence factors, all the results that contained *p* value < 0.05 were regarded as statistically significant. We processed data using IBM SPSS software version 27.0 (IBM company, North Castle, Armonk, NY, USA).

## 3. Results

### 3.1. Routine Changes

**Figure 1:**
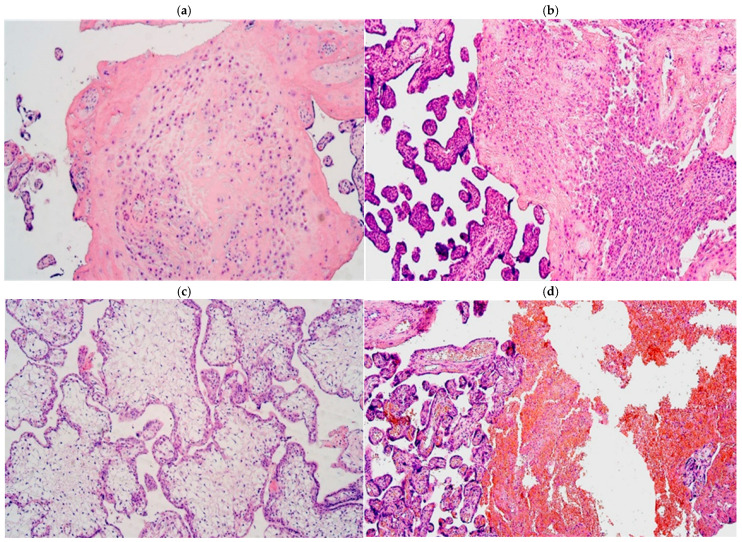
(**a**–**d**) Routine staining micrographs of placental tissue samples: (**a**) numerous Hofbauer cells in 31 gestational week placenta; (**b**) 28 gestational week placenta containing inflammatory cells; (**c**) 31 gestational week edematic placenta; (**d**) 28 gestational week placenta with blood vessel plethora. Hematoxylin and eosin, ×100 for all the slides.

Every examined placental tissue revealed numerous accumulations of Hofbauer cells (Figure 1a). An abundant number of inflammatory cell infiltration in a few areas was noticed in almost all examined placental tissue samples except in control samples, where there was no significant inflammatory cell infiltration observed (Figure 1b). Edema was observed in two samples (Figure 1c), and a plethora in five samples (Figure 1d), although all these changes did not differ between the gestational weeks.

### 3.2. Immunohistochemistry

#### 3.2.1. Distressed Placental Tissue Samples

**Figure 2:**
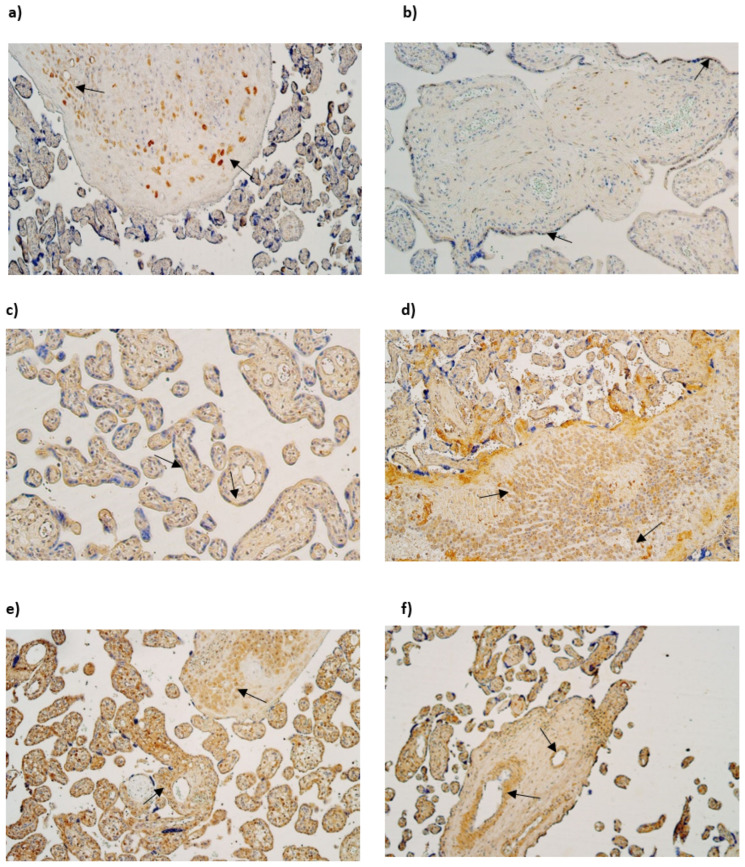
(**a**–**f**) IMH micrographs of placental tissue samples with different gestational weeks: (**a**) moderate number of HBD-2-positive Hofbauer cells in 28 gestational week placenta (arrows). HBD2 IMH, ×100; (**b**) few to moderate HBD-2-positive cytotrophoblast cells in tertiary villy in 31 gestational week placenta (arrows). HBD-2 IMH, ×100; (**c**) moderate number of HBD3-positive cytotrophoblast cells in tertiary villy in 28 gestational week placenta (arrows). HBD-3 IMH, ×100; (**d**) numerous to abundant HBD-3-positive Hofbauer cells in 40 gestational week placenta (arrows) HBD-3 IMH, ×100; (**e**) numerous to abundant HBD-4-positive extraembryonic mesodermal and Hofbauer cells in 28 gestational week placenta (arrows). HBD-4 IMH × 100; (**f**) abundant number of HBD-4-positive endothelial cells of tertiary villy in 40 gestational week placenta (arrows). HBD-4 IMH, ×100.

The median number of HBD-2-immunopositive cytotrophoblasts ranged from none (0) to few (+) in gestational weeks 28, 31, and 40. Similarly, the median number of HBD-2-immunopositive endothelial cells varied from none (0) to occasional (0/+) in the samples examined across different gestational weeks. However, the median number of HBD-2-immunoreactive extraembryonic mesodermal cells ranged from none (0) to moderate (++), and HBD-2-immunoreactive Hofbauer cells ranged from few (+) to moderate (++) (Table 3, Figure 2a,b).

The median number of HBD-3-immunopositive cytotrophoblasts had a variation from moderate (++) to numerous (+++) in examined different gestational week samples. The median number of HBD-3-immunoreactive endothelial cells in gestational weeks 28 and 31 was few (+), although in week 40 it was moderate (++). The median number of HBD-3-immunopositive extraembryonic mesodermal cells varied from moderate (++) to abundant (++++) in different gestational week samples, although the median number of HBD-3-immunoreactive Hofbauer cells in week 28 was few to moderate (+/++) and in week 31 and 40—moderate (++) (Table 3, Figure 2c,d).

The median number of HBD-4-immunoreactive cytotrophoblasts varied from the lack (0) to moderate (++) in gestational weeks 28, 31 and 40. The median number of HBD-4-immunopositive endothelial cells in weeks 28 and 31 was few (+), although in week 40 it was moderate to numerous (++/+++). In contrast the median number of HBD4-immunoreactive extraembryonic mesodermal cells was moderate to numerous (++/+++) in weeks 28, 31 and 40. The median number of HBD-4-immunoreactive Hofbauer cells in 28 and 31 gestational week samples was numerous to abundant (+++/++++), although in gestational week 40 median number was numerous (+++) (Table 3, Figure 2e,f).

**Figure 3:**
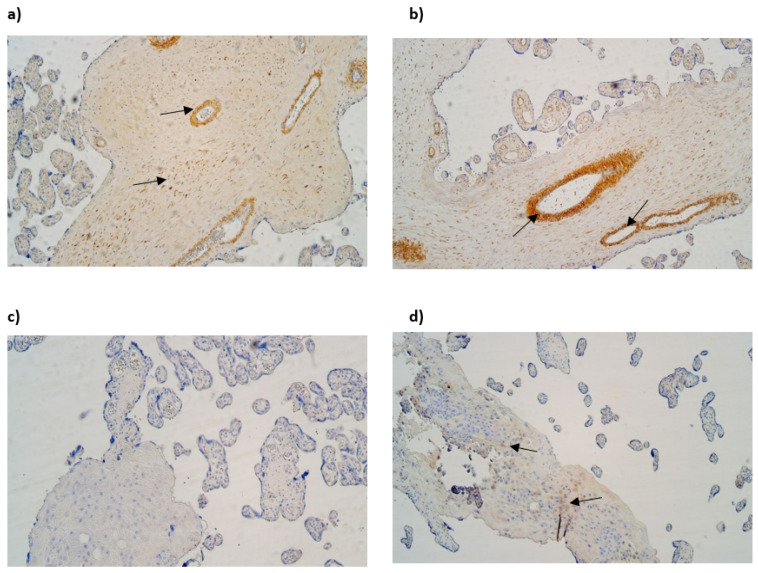
(**a**–**d**) IMH micrographs of placental tissue of different gestational weeks: (**a**) Numerous to abundant positive NF-κB cells in endothelium and extraembryonic mesoderm in 31 gestational week placenta (arrows). NF-kB IMH, ×100; (**b**) abundance of NF-kB-positive endothelial cells in 40 gestational week placenta (arrows). NF-κB IMH, ×100; (**c**) absence of LL-37-positive cells in placental tissue in 28 gestational week placenta. LL-37 IMH, ×100; (**d**) few to moderate LL-37-positive Hofbauer cells in 40 gestational week placenta (arrows). LL-37 IMH, ×100.

NF-κB was not discovered in cytotrophoblasts. The median number of NF-κB-immunoreactive endothelial cells varied from moderate to numerous (++/+++) in gestational weeks 28, 31 and 40. The median number of NF-κB-immunoreactive extraembryonic mesodermal cells varied from few to moderate (+/++) in examined samples and the median number of immunoreactive Hofbauer cells was occasional (0/+) in gestational week 28, although in gestational week 31 and 40, it was few (+) (Table 3, Figure 3a,b).

Cathelicidin (LL-37) immunopositivity was observed in Hofbauer cells only, although only in samples of gestation week 40 and it had a variety of the lack (0) to few (+) positive cells (Figure 3c,d).

**Figure 4:**
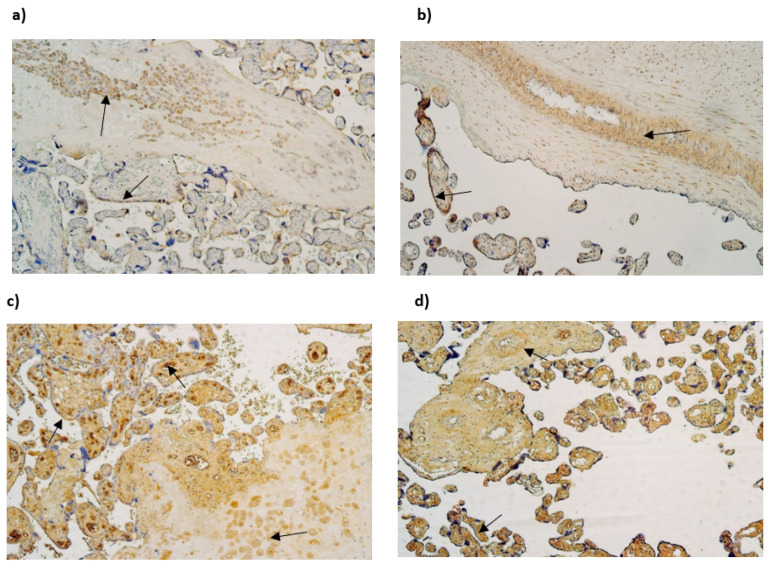
(**a**–**d**) IMH micrographs of placental tissue of different gestational weeks; (**a**) moderate to numerous number of HSP60-positive Hofbauer and moderate number HSP60-positive cytotrophoblasts in 31 gestational week placenta (arrows). HSP60 IMH × 100; (**b**) abundant number of HSP60-positive endothelial and cytotrophoblast cells in placental tissue in 40 gestational week placenta (arrows). HSP60 IMH × 100; (**c**) numerous to abundant number of IL-10 in 28 gestational week placenta (arrows). IL-10 IMH, ×100; (**d**) moderate to numerous number of IL-10-positive endothelial cells and numerous to abundant number of IL-10-positive extraembryonic mesodermal cells in 40 gestational week placenta (arrows). IL-10 IMH, ×100.

The median number of HSP60-immunopositive cytotrophoblasts and Hofbauer cells in all examined samples was moderate (++). In contrast, the median number of HSP60-immunoreactive endothelial cells was occasional (0/+) in gestational weeks 28 and 31, while in week 40 it was few to moderate (+/++). The median number of HSP60-immunopositive extraembryonic cells in week 28 was occasional (0/+), while in weeks 31 and 40, it was few (+) (Table 3, Figure 4a,b).

The median number of IL-10-immunopositive cytotrophoblasts was moderate (++) in samples of all gestational weeks. Interestingly, the median number of immunoreactive endothelial and extraembryonic mesodermal cells was moderate to numerous (++/+++) in weeks 28 and 40 and numerous (+++) in weeks 31. The median number of IL-10-immunoreactive Hofbauer cells in weeks 28 was moderate (++), although, in weeks 31 and 40, it was numerous (+++) (Table 3, Figure 4c,d).

#### 3.2.2. Control Placental Tissue Samples

**Figure 5:**
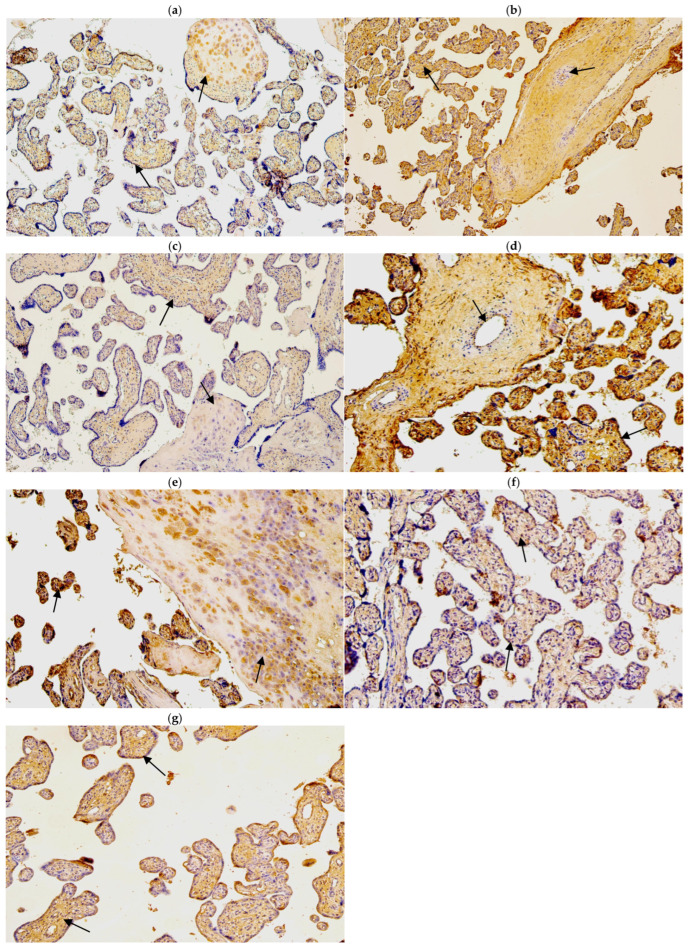
(**a**–**g**). IMH micrographs of control samples of placental tissue; (**a**) numerous number of HBD-2-positive Hofbauer and few number of HBD-2-positive cytotrophoblasts in 40 gestational week placenta (arrows). HBD-2 IMH, ×100; (**b**) numerous to abundant number of HBD-3-positive extraembryonic mesodermal cells and few HBD-3-positive endothelial cells in 40 gestational week placenta (arrows). HBD-3 IMH, ×100; (**c**) moderate number of HBD-4-positive extraembryonic cells and few number of HBD-4-positive Hofbauer cells in 40 gestational week placenta (arrows). HBD-4 IMH, ×100; (**d**) moderate number of NF-κB-positive endothelial cells and abundant number of NF-κB-positive cytotrophoblasts in 40 gestational week placenta (arrows). NF-κB IMH, ×100; (**e**) numerous to abundant number of IL-10-positive Hofbauer cells and numerous number of IL-10-positive cytotrophoblasts in 40 gestational week placenta (arrows). IL-10 IMH, ×100; (**f**) moderate number of HSP60-positive cytotrophoblasts and extraembryonic mesodermal cells in 40 gestational week placenta (arrows). HSP60 IMH, ×100; (**g**) numerous number of LL-37-positive extraembryonic mesodermal cells and moderate number of LL-37-positive cytotrophoblasts (arrows). LL-37 IMH, ×100.

In control samples of gestational week 40, the median number of HBD-2-immunopositive cytotrophoblasts was few (+), the median number of HBD-2-immunopositive endothelial cells was occasional (0/+), the median number of HBD-2-immunoreactive extraembryonic mesodermal was few to moderate (+/++), and the median number of HBD-2-immunoreactive Hofbauer cells was moderate (++) (Table 4, Figure 5a).

In control samples of gestational week 40, the median number of HBD-3-immunopositive cytotrophoblasts was moderate to numerous (++/+++). The median number of HBD-3-immunoreactive endothelial cells was few (+). The median number of HBD-3-immunopositive extraembryonic mesodermal cells was numerous to abundant (++/+++), although the median number of HBD-3-immunoreactive Hofbauer cells was few to moderate (+/++) (Table 4, Figure 5b).

In control samples of gestational week 40, the expression of HBD-4 in cytotrophoblasts was occasional (0/+), and the median number of HBD-4-immunopositive endothelial cells and extraembryonic mesodermal cells was few (+). In contrast, the median number of HBD-4-immunoreactive Hofbauer cells was moderate (++) (Table 4, Figure 5c).

The median number of NF-κB-positive cytotrophoblasts was numerous (+++) The median number of NF-κB-immunoreactive extraembryonic mesodermal cells was moderate (++), although the median number of NF-κB-immunoreactive endothelial cells and Hofbauer cells was few to moderate (+/++) (Table 4, Figure 5d).

The median number of IL-10-immunopositive cytotrophoblasts was moderate (++). Interestingly, the median number of immunoreactive extraembryonic mesodermal and Hofbauer cells was moderate to numerous (++/+++), although the median number of IL-10-immunopositive endothelial cells was few (+) (Table 4, Figure 5e).

The median number of HSP60-immunopositive cytotrophoblasts, extraembryonic mesodermal cells, and Hofbauer cells was moderate (++). The median number of HSP60-immunoreactive endothelial cells was few (+) (Table 4, Figure 5f).

The median number of Cathelicidin (LL-37)-immunopositive cytotrophoblasts and Hofbauer cells was moderate (++), the median number of Cathelicidin (LL-37)-immunoreactive endothelial cells occasional (0/+), and the median number of immunopositive extraembryonic mesodermal cells was moderate to numerous (++/+++) (Table 4, Figure 5g).

**Figure 6:**
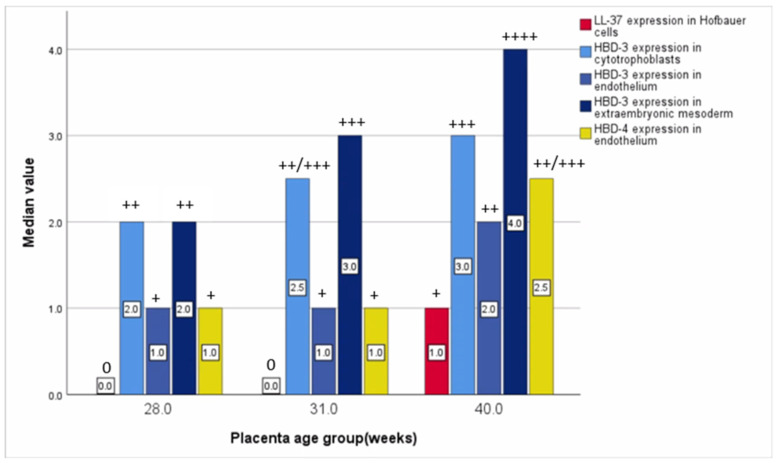
Relative median number of statistically significant evaluated defence factors based on placenta’s gestational weeks.

Statistically significant differences between distressed placental tissue samples were evaluated using the Kruskal–Wallis test between different gestational week placental tissue samples in LL-37-positive Hofbauer cells, HBD-3-positive cytotrophoblasts, endothelial cells, extraembryonic mesodermal cells, and HBD-4-containing endothelial cells. The relative median number of LL-37-immunopositive cells in gestational weeks 28 and 31 was 0 (0), although in gestational week 40, it was 1 (+). The relative median number of HBD-3-immunopositive cytotrophoblasts in gestational week 28 was 2 (++), and, in week 31, it was 2.5 (++/+++), although, in week 40, it was 3 (+++). The relative median number of HBD-3-immunoreactive endothelial cells in gestational weeks 28 and 31 was 1 (+), although in week 31, it was 2 (++). The relative median number of HBD-3-immunopositive extraembryonic mesodermal cells in gestational week 28 was 2 (++), 3 in week 31 (+++), and 4 in week 40 (++++). The relative median number of HBD-4-immunoreactive endothelial cells in weeks 28 and 31 was 1 (+), although in week 40, it was 2.5 (++/+++) (Table 5, Figure 6).

Statistically significant differences between control placental tissue samples and distressed placental tissue samples of gestational week 40 were found using the Mann–Whitney U test in NF-κB-positive cytotrophoblasts and endothelial cells, HBD-2-containing extraembryonic mesodermal cells, HBD-4-containing extraembryonic mesodermal and endothelial cells, and LL-37-positive extraembryonic mesodermal cells and cytotrophoblasts. Furthermore, statistically significant differences between IL-10-positive endothelial cells and HSP60-positive extraembryonic mesodermal cells were found. The relative median number of NF-κB-immunopositive cytotrophoblasts in distressed placental tissue of gestational week 40 was 0 (0), although, in control samples of gestational week 40, it was 3 (+++). The relative median number of NF-κB-immunopositive endothelial cells in distressed placental tissue of gestational week 40 was 3 (+++), although in control samples, it was 1.5 (+/++). Furthermore, the relative median number of HBD-2-immunopositive extraembryonic mesodermal cells in distressed placental tissue of gestational week 40 was 0 (0), although, in control samples, it was 1.5 (+/++). The relative median number of HBD-4-immunopositive extraembryonic mesodermal cells and endothelial cells in distressed placental tissue of gestational week 40 was 2.5 (++/+++), which is in contrast to control samples, where both relative median numbers of the expression in these structures were 1 (+). In addition, in distressed placental tissue of gestational week 40, LL-37-positive extraembryonic mesodermal cells and cytotrophoblasts were not found, although the relative median numbers of LL-37-positive extraembryonic mesodermal cells and cytotrophoblasts in control samples were 2 (++) and 1.5 (+/++), respectively. The relative median number of IL-10-positive endothelial cells in distressed placental tissue samples of gestational week 40 was 2.5 (++/+++), although, in control samples, it was 1 (+). The relative median number of HSP60-immunopositive extraembryonic mesodermal cells in distressed placental tissue samples of gestational week 40 was 1 (+), although, in control samples, it was 2 (++) (Table 6).

**Figure 7:**
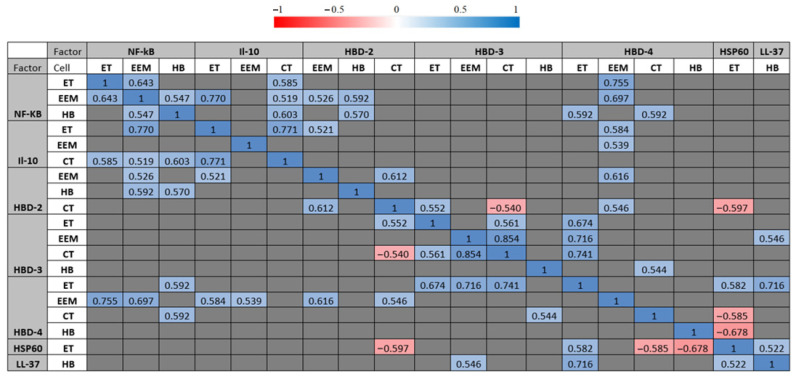
Statistically significant correlation values between examined factors (*p* < 0.05). Spearman’s rank correlation test was used for analysis. Defence factors with one or more statistically significant correlations are shown. Correlation values are coloured based on correlation legend. Correlation legend is shown on top. Red colours indicate a negative correlation between defence factors, while blue colours show a positive correlation between defence factors. The saturation of the colour indicates stronger (negative or positive) Spearman’s rank correlation coefficients. Correlation values that were not statistically significant are marked as grey cells.

Correlation analysis revealed multiple statistically significant correlations between the factors of immunopositive cells in distressed placental tissue. A statistically notable and very strong positive correlation (R = 0.854; *p* = <0.001) was calculated between HBD-3-immunoreactive cytotrophoblasts and HBD-3-immunoreactive extraembryonic mesodermal cells. Statistically notable and strong positive correlations (R = 0.6–0.8) were evaluated in multiple immunopositive cells between HBD-2-immunoreactive cytotrophoblasts and HBD-2-immunoreactive extraembryonic mesodermal cells (R = 0.612; *p* = 0.015); between HBD-2-immunopositive extraembryonic mesodermal cells and HBD-4-immunopositive extraembryonic mesodermal cells (R = 0.616; *p* = 0.014); between HBD-3-immunoreactive cytotrophoblasts and HBD-4-immunopositive endothelial cells (R = 0.741; *p* = 0.002); between HBD-3-immunoreactive extraembryonic mesodermal cells and HBD-4-immunoreactive endothelial cells (R = 0.716; *p* = 0.003); between HBD-4-immunopositive endothelial cells and LL-37-immunopositive Hofbauer cells (R = 0.716; *p* = 0.003); between NF-κB-immunoreactive endothelial cells and NF-κB-immunoreactive extraembryonic mesodermal cells (R = 0.643; *p* = 0.010); between NF-κB-immunoreactive Hofbauer cells and IL-10-immunoreactive cytotrophoblasts (R = 0.603; *p* = 0.017); between NF-κB-immunopositive endothelial cells and HBD-4-immunopositive extraembryonic mesodermal cells (R = 0.755; *p* = 0.001); between NF-κB-immunoreactive extraembryonic mesodermal cells and IL-10-immunoreactive endothelial cells (R = 0.770; *p* = <0.001); between NF-κB-immunopositive extraembryonic mesodermal cells and HBD-4-immunopositive extraembryonic mesodermal cells (R = 0.697; *p* = 0.004); and between IL-10-immunoreactive extraembryonic mesodermal cells and IL-10-immunoreactive cytotrophoblasts (R = 0.771; *p* = <0.001). Interestingly, a strong negative correlation (R = −0.678; *p* = 0.005) was calculated between HSP60-positive endothelial cells and HBD-4-positive Hofbauer cells (Table 7, Figure 7).

## 4. Discussion

In this study, HBD-2, HBD-3 and HBD-4 expression was observed in all distressed placental structures. However, we found that the relative number of HBD-2-positive cytotrophoblasts and extraembryonic mesodermal and endothelial cells had a tendency to decrease with higher gestational weeks. In contrast, there were statistically significant differences in the relative number of HBD-3-positive extraembryonic mesodermal and endothelial cells that increased with higher gestational weeks. According to these findings, we suspect that HBD-2 expression is not essential for placenta maturation, and it is substituted by an increase in selective HBD-3 expression mainly in the endothelium and extraembryonic mesoderm. It is known that in placental tissue, fungi and bacteria act as stimuli for HBD-2 expression [22], and, during early pregnancy, low levels of HBD-2 suggest the presence of a poor vaginal environment, thus increasing the possibility of developing (premature rupture of membranes (PROM)), affecting placental tissue as well [20]. Therefore, in this study, the higher expression of HBD-2 in gestational weeks 28 and 31 could be linked with the absence of a poor vaginal environment and probably the presence of pathogenic microorganisms. Our findings that show HBD-3 expression increases with higher gestational weeks are similar to the findings by Anne E King et al. in 2003 [23], which showed that the amniotic fluid concentrations of HBD-3 were higher in women that have spontaneous labour at term; moreover, HBD-3 expression does not change with gestational age in normal pregnancies [23], which is contradictory to our findings of various expression levels in different gestational weeks groups. The expression of HBD-4 between three gestational weeks groups varied in placental structures, indicating the presence of some individual factor affecting the expression, although no significant differences between these groups were noted. In the extraembryonic mesoderm and Hofbauer cells, it was consistently high throughout all three gestational weeks groups. HBD-4 expression showed a tendency to decrease in cytotrophoblasts in higher gestational weeks, although a statistically significant increase in HBD-4-positive endothelial cells with higher gestational weeks was observed. These are completely new findings and the variation in expression could be linked with circumstances of placenta distress. It is known that compared with other β -defensins, which are associated with strong chemotaxis, HBD-4 does not induce CCR6-mediated chemotaxis [51], although HBD-4 can induce a significant migration of monocytes [52]. The overall expression of HBD (2,3,4) in placental structures was selective. HBD-3 and HBD-4 were mainly produced in extraembryonic mesoderm and Hofbauer cells, although HBD-2 was largely produced only in Hofbauer cells. Interestingly, a very strong correlation was calculated between HBD-3-immunoreactive cytotrophoblasts and HBD-3-immunoreactive extraembryonic mesodermal cells, which reveals that HBD-3 function is strongly linked with various placental structures.

In this study, high IL-10 expression was observed in all distressed placental structures, and no statistically significant differences in IL-10 expression were observed between the groups. It was consistent without great variation throughout all gestational weeks, indicating that IL-10 activity in the placenta is consistent during pregnancy. We suspect that IL-10 is important in sustaining the successful growth of the placenta throughout pregnancy. Different studies suggest that decidual macrophages produce Il-10 and are the major source of IL-10; apart from the location, there are no specific markers to distinguish decididual macrophages from Hofbauer cells [47,53]. There are reports that IL-10 is a critical molecule for successful pregnancy outcomes; in addition, in contrast to this study, where IL-10 expression was consistent between all three gestational week groups, the placental expression of IL-10 was found to be reduced in spontaneous abortions and preterm births [43].

In this study, LL-37 expression in distressed placental structures was detected only in a few Hofbauer cells in gestational week 40 placenta, although it was a statistically significant result compared with earlier gestational weeks. Also, it is known that LL-37 expression is higher in fetal membranes and myometrium after term labour. LL-37 also induces proinflammatory and pro-labour mediators via the MyD88/NF-kB pathway [54]. Higher LL-37 presence in higher gestational weeks placenta and only in Hofbauer cells may indicate that LL-37 is not an important component in protective mechanisms in the placenta; however, a study in 2015 by Ratana Lim et al. [54] showed that in vitro LL-37 boosts the immunosuppressive function of placenta-derived mesenchymal stromal cells and modulates TLR3 expression, promoting higher levels of anti-inflammatory factors. A study in 2016 by Martha Oliveira-Bravo et al. showed that increased levels of LL-37 can lead to an increased expression of IL-10; however, in our study, there was no statistically significant correlation between LL-37 and IL-10 expression [55].

The expression of NF-kB varied between distressed placental structures. A high relative number of NF-kB-positive cells was seen mainly in the endothelium, and it showed a tendency to increase in higher gestational weeks; therefore, we suspect that NF-kB plays an important role in advanced stages of pregnancy, modulating vascular function in the placenta. These findings are supported by a study in 2020 by Armistead et al. [56], which showed that NF-kB is highly expressed in the placentas of women with pre-eclampsia where there is vascular dysfunction. It is also known that there are molecules that can activate NF-kB, such as damage-associated molecular patterns (DAMPs). They are molecules released when there is cellular stress, and they activate NF-KB-1 through TLR receptor pathways, inducing proinflammatory cascades [16,56,57]. NF-kB is associated with preterm birth when there is an interaction with activator protein 1 (AP-1) [56]. Extravillous trophoblast invasion is partially regulated by NF-kB [55]. Epithelial-to-mesenchymal transition is regulated by NF-kB and it plays a significant role in extravillous trophoblasts [58]. Our findings of increased expression in higher gestational week placenta can be supported by a study in 2018 by Sakowicz [38], which showed that higher NF-kB expression is seen in the third trimester of normal pregnancy in the decididua, where this factor induces cervical ripening and the degradation of the extracellular matrix to initiate the rupture of placental membranes. Lower NF-Kb expression was seen in the extraembryonic mesoderm and Hofbauer cells throughout all three gestational weeks groups. These findings indicate NF-kB’s capabilities to support placenta function during pregnancy. A lack of NF-kB expression was observed in cytotrophoblasts across all gestational week placentas.

A higher expression of HSP60 was steadily seen in the cytotrophoblasts and Hofbauer cells, although expression levels did not change between the three gestational week groups. A lower expression of HSP60 was seen in the endothelium and extraembryonic mesoderm and the expression levels were unvarying in the three gestational weeks groups as well. Interestingly, a strong negative correlation was calculated between HSP60-positive endothelial cells and HBD-4-positive Hofbauer cells, which shows possible negative feedback loop actions between these factors. The findings in this study are newly discovered, and we suspect that HSP60 plays an important role in supporting protective mechanisms in the placenta during pregnancy. It is also acknowledged that in pregnancy, HSP60 plays a role in inducing the synthesis of steroid hormones, particularly progesterone synthesis. Preterm premature rupture of the membranes and spontaneous preterm labour are associated with the dysregulated expression of HSP60 and other HSPs, and expression is found to be severely altered [40]. One of the mechanisms of action for HSP60 includes the ability to interact with HSP70 to form an HSP60-HSP70 complex, and this complex allows the transport of proteins [59].

There were multiple statistically significant differences in factor expression in various placental structures between distressed placental tissue samples of 40 gestational weeks and control samples at the same gestational week. All factors except HBD-3 had a significant change in expression in at least one of the placental structures. Control samples showed a statistically significantly higher expression of NF-kB and LL-37 in cytotrophoblasts and HBD-2, HSP60, and LL-37 in the extraembryonic mesoderm. In contrast, control samples showed a statistically significant lower expression of NF-kB, IL-10, and HBD-4 in the endothelium and HBD-4 in the extraembryonic mesoderm.

With everything taken into account, the expression of HBD-3 and NF-kB was observed to a greater extent in higher gestational weeks with distressed placental structures, which could indicate the role of these factors in later periods of pregnancy. However, the expression of IL-10 and HSP60 was more constant throughout all three gestational weeks and shows the role of these factors throughout the entire period of pregnancy. In addition, the expression of HBD-2 and HBD-4 had great variety between different gestational weeks, which may indicate that there is another factor influencing the expression of these defence factors.

### Limitations and Future Perspectives

This study has limitations. There was a small number of placental tissue samples, and a higher number of samples could provide more illustrative results. Additionally, there was limited demographic and clinical data about participants, and more in-depth information could be helpful in determining possible additional reasons for the different results regarding defence factor expression levels in placental tissue. Furthermore, it is difficult to gather placental tissues and obtain control samples due to ethical reasons. The next issue is the evaluation of the concentration of the abovementioned factors by ELISA, which could give additional information about the common levels of them in the placenta at different gestational times. Finally, the combination of all the abovementioned factors with other cytokines and remodelling factor expression might give a more complete picture of specific molecular events in the placenta at different developmental times.

As for future perspectives, there is still the possibility of discovering defence factors and their role and functional significance in placental tissue; understanding their role could have clinical implications for immune response processes and may possibly affect pregnancy outcomes. Our study could be expanded in the future by observing levels of defence factors during different gestational week placental tissue and how they interact with other processes occurring in the protection mechanisms of the placenta, which could help understand the functional significance of these defence factors.

## 5. Conclusions

The persistence of Hofbauer cell accumulations underlines the growing significance of placental macrophages in placental protection.

The expression of positive defence factors and an increase in the expression of tissue protection factors (HBD-3, LL-37, HBD-4) in later gestational weeks may indicate these factors as the most significant protectors of the placenta in the ontogenetic aspect.

The high number of statistically significant positive and negative correlations between factor-positive cells show a strong network to sustain distressed placental growth and therefore pregnancy.

## Figures and Tables

**Table 1:** Characteristics of placental tissue samples from different mothers with different delivery weeks and possible associated problems.

No.	Female Age	Graviditas	Partus	Delivery Week/Associated Problems
**28 weeks**				
7	36	I	I	28, spontaneous preterm delivery, body mass index before the actual pregnancy (BMI) = 32.3
14	20	I	I	28, spontaneous preterm delivery, BMI = 28.0
19	31	II	II	28, spontaneous preterm delivery, placental abruption, uterus bicornis, BMI = 21.0
20	26	I	I	28, spontaneous preterm delivery, preterm premature rupture of membranes, BMI = 25.6
24	36	II	I	28, spontaneous preterm delivery, preterm premature rupture of membranes, BMI = 23.8
**31 weeks**				
22	36	II	II	31, partum premature operationis, BMI = 19.7
23	39	VI	II	31, partum premature operationis, placental abruption, BMI = 19.4
26	36	I	I	31, spontaneous preterm delivery, BMI = 36.6
32	28	IV	I	31, spontaneous preterm delivery, gemelli, umbilical vein thrombosis, BMI = 19.9
33	28	IV	I	31, spontaneous preterm delivery, gemelli, umbilical vein thrombosis, BMI = 19.9
**40 weeks**				
2	39	IV	II	40, partus mature, acute fetal distress, BMI = 21.0
9	27	III	III	40, partus mature, acute fetal distress, BMI = 25.6
10	23	I	I	40, partus mature operationis, acute fetal distress, BMI = 18.7
13	31	III	I	40, partus mature operationis, acute fetal distress, intranatal dead fetus, BMI = 28.0
16	22	I	I	40, partus mature, acute fetal distress, BMI = 23.8

**Table 2:** Characteristics of placental tissue control samples from mothers with delivery week 40.

No.	Female Age	Graviditas	Partus	Delivery Week
5	27	I	I	40, partus mature, BMI = 22.6
0017	37	IV	II	40, partus mature, BMI = 30.5
0081	29	II	II	40, partus mature, BMI = 26.4
0107	42	III	I	40, partus mature, BMI = 20.4
8423	32	III	III	40, partus mature, BMI = 20.3

**Table 3:** Median relative number of HBD-2-, HBD-3-, HBD-4-, NF-κB-, HSP60-, and IL-10-positive structures in distressed placental tissue across different gestational weeks.

**Factor**	**HBD2**				**HBD3**			
**Cell Type**	**CT**	**ET**	**EEM**	**HB**	**CT**	**ET**	**EEM**	**HB**
28 weeks	+	0/+	++	+/++	++	+	++	+/++
31 weeks	+	0/+	+/++	+/++	++/+++	+	+++	++
40 weeks	0	0	0	+/++	+++	++	++++	++
**Factor**	**HBD4**				**Il-10**			
**Cell Type**	**CT**	**ET**	**EEM**	**HB**	**CT**	**ET**	**EEM**	**HB**
28 weeks	++	+	++/+++	+++/++++	++	++/+++	++/+++	++
31 weeks	+	+	++/+++	+++/++++	++	+++	+++	+++
40 weeks	0/+	++/+++	++/+++	+++	++	++/+++	++/+++	+++
**Factor**	**NF-kB**				**HSP60**			
**Cell Type**	**CT**	**ET**	**EEM**	**HB**	**CT**	**ET**	**EEM**	**HB**
28 weeks	0	++	+	0/+	++	0/+	0/+	++
31 weeks	0	++/+++	++	+	++	0/+	+	++
40 weeks	0	+++	+/++	+	++	+/++	+	++
**Factor**	**LL-37**							
**Cell Type**	**CT**	**ET**	**EEM**	**HB**				
28 weeks	0	0	0	0				
31 weeks	0	0	0	0				
40 weeks	0	0	0	+				

Abbreviations: HBD2—human β defensin 2; HBD3—human β defensin 3; HBD4—human β defensin 4; IL-10—interleukin NF-kB—Nuclear factor kappa B; HSP60—heat shock protein 60; CT—cytotrophoblasts, ET—endothelial cell; EEM—extraembryonic mesodermal cell; HB—Hofbauer cell; 0—no positive cells; 0/+—occasional positive cells; +—few positive cells; +/++—few to moderate positive cells; ++—moderate number of positive cells; ++/+++—moderate to numerous positive cells; +++—numerous positive cells in observed visual field; +++/++++—numerous to abundant positive cells; ++++—abundant positive cells in the visual field.

**Table 4:** Median relative number of HBD-2-, HBD-3-, HBD-4-, NF-κB-, HSP60-, and IL-10-positive structures in control placental tissue.

**Factor**	**HBD2**				**HBD3**			
**Cell Type**	**CT**	**ET**	**EEM**	**HB**	**CT**	**ET**	**EEM**	**HB**
Control	+	0/+	+/++	++	++/+++	+	+++/++++	+/++
**Factor**	**HBD4**				**Il-10**			
**Cell Type**	**CT**	**ET**	**EEM**	**HB**	**CT**	**ET**	**EEM**	**HB**
Control	0/+	+	+	++	++	+	++/+++	++/+++
**Factor**	**NF-kB**				**HSP60**			
**Cell Type**	**CT**	**ET**	**EEM**	**HB**	**CT**	**ET**	**EEM**	**HB**
Control	+++	+/++	++	+/++	++	+	++	++
**Factor**	**LL-37**							
**Cell Type**	**CT**	**ET**	**EEM**	**HB**				
Control	+/++	0/+	++	+/++				

Abbreviations: HBD2—human β defensin 2; HBD3—human β defensin 3; HBD4—human β defensin 4; IL-10—interleukin 10 NF-kB—nuclear factor kappa B; HSP60—heat shock protein 60; CT—cytotrophoblast, ET—endothelial cell; EEM—extraembryonic mesodermal cell, HB—Hofbauer cell; 0/+—occasional positive cells; +—few positive cells; +/++—few to moderate positive cells; ++—moderate number of positive cells; ++/+++—moderate to numerous positive cells; +++—numerous positive cells in observed visual field; +++/++++—numerous to abundant positive cells.

**Table 5:** Statistically significant differences between relative number of defence factors in placental tissue samples of placentas across different gestational weeks.

	LL-37 in Hofbauer Cells	HBD-3 in Cytotrophoblasts	HBD-3 in Endothelium	HBD-3 in Extraembryonic Mesoderm	HBD-4 in Endothelium
Kruskal–Wallis	7	9.866	7.425	10.975	13.462
*p* value	0.03	0.007	0.024	0.004	0.001

**Table 6:** Statistically significant differences between relative number of defence factors comparing distressed placental tissue samples of gestational week 40 and control placental tissue samples of gestational week 40.

	NF-κB in CT	NF-κB in ET	HBD-2 in EEM	HSP60 in EEM	IL-10 in ET	LL-37 in EEM	LL-37 in CT	HBD-4 in EEM	HBD-4 in ET	HBD-2 in EEM
Mann–Whitney U	0	3	3	3.5	1.5	0	0	0	0	3
*p* value	<0.005	0.043	0.037	0.050	0.017	<0.005	0.004	0.006	0.006	0.037

Abbreviations: ET—endothelial cell; EEM—extraembryonic mesodermal cell; CT—cytotrophoblast cell; NF-κB—Nuclear factor kappa B; IL-10—Interleukin-10; HBD-2—human β defensin 2; HBD-4—human β defensin 4; HSP60– heat shock protein 60; LL-37—Cathelicidin LL-37.

**Table 7:** Statistically notable correlations between factors in placental structures.

Strength of Correlation	Factor 1	Factor 2	R	*p*
**Positive Correlations**				
Very strong (0.8–1.0)	HBD-3 CT	HBD-3 EEM	0.854	<0.001
Strong (0.6–0.8)	HBD-2 CT	HBD-2 EEM	0.612	0.015
	HBD-2 EEM	HBD-4 EEM	0.616	0.014
	HBD-3 CT	HBD-4 ET	0.741	0.002
	HBD-3 EEM	HBD-4 ET	0.716	0.003
	HBD-4 ET	LL-37 HB	0.716	0.003
	NF-κB ET	NF-κB EEM	0.643	0.010
	NF-κB HB	IL-10 CT	0.603	0.017
	NF-κB ET	HBD-4 EEM	0.755	0.001
	NF-κB EEM	IL-10 ET	0.770	<0.001
	NF-κB EEM	HBD-4 EEM	0.697	0.004
	IL-10 EEM	IL-10 CT	0.771	<0.001
**Negative Correlation**				
Strong (0.6–0.8)	HBD-4 HB	HSP60 ET	−0.678	0.005

Abbreviations: ET—endothelial cell; EEM—extraembryonic mesodermal cell; CT—cytotrophoblast cell; HB—Hofbauer cell; NF-κB—Nuclear factor kappa B; IL-10—Interleukin-10; HBD-2—human β defensin 2; HBD-3—human β defensin 3; HBD-4—human β defensin 4; HSP60– heat shock protein 60; LL-37—Cathelicidin LL-37; R—Spearman’s rank correlation coefficient, *p*—*p* value.

## Data Availability

All the data presented in this study are available upon request from the corresponding author.
